# Modified clays alter diversity and respiration profile of microorganisms in long‐term hydrocarbon and metal co‐contaminated soil

**DOI:** 10.1111/1751-7915.13510

**Published:** 2019-11-11

**Authors:** Bhabananda Biswas, Albert L. Juhasz, Mohammad Mahmudur Rahman, Ravi Naidu

**Affiliations:** ^1^ Future Industries Institute University of South Australia Mawson Lakes SA 5085 Australia; ^2^ Cooperative Research Centre for Contamination Assessment and Remediation of the Environment (CRC CARE) The University of Newcastle ATC Building Callaghan NSW 2308 Australia; ^3^ Global Centre for Environmental Remediation (GCER) The University of Newcastle Callaghan NSW 2308 Australia

## Abstract

Clays and surfactant‐modified clays (organoclays) are becoming popular as pollutant sorbents due to their high reactivity and low‐cost availability. However, the lack of field testing and data on ecotoxicity limits their application. Considering such aspects, this study assessed the impact of clay amendments to polycyclic aromatic hydrocarbons (PAHs)/cadmium (Cd)‐contaminated soil on microbial respiration profiles (active vs. inactive cells) using redox staining and the relative abundance and diversity of bacteria and archaea. These clay products are bentonite, cationic surfactant‐modified bentonite and palmitic acid‐grafted surfactant‐modified bentonite). After 70 days, the addition of bentonite and its modified forms altered microbial community structure mainly among dominant groups (*Actinobacteria*, *Proteobacteria*, *Firmicutes* and *Chloroflexi*) with effects varying depending on material loading to soil. Among amendments, fatty acid (palmitic acid) tailored cationic surfactant‐modified bentonite proved to be microbial growth supportive and significantly increased the number of respiration‐active microbial cells by 5% at a low dose of material (e.g. 1%). Even at high dose (5%), the similarity index using operational taxonomic units (OTUs) also indicates that this modified organoclay‐mixed soil provided only slightly different environment than control soil, and therefore, it could offer more biocompatibility than its counterpart organoclay at similar dose (e.g. cationic surfactant‐modified bentonite). This study promotes designing ‘eco‐safe’ clay‐based sorbents for environmental remediation.

## Graphical abstract

Environmental application of clay and modified clays are promising; however, there might be potential impact attributed to the soil amendments with these materials. This study presents that surfactant‐modified clay and their fatty acid tailored products could change microbial diversity and respiration profile depending on the dose applied to the soil.

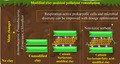

## Introduction

Material‐based remediation of soil contaminated with PAHs and metals (e.g. Cd) is well documented at laboratory and field scale (Lohmann *et al.*, 2005; Qu *et al.*, 2008; Chen and Yuan, 2011; Zhang *et al.*, 2013). In most cases, sorption of contaminants is the focus, but the impact of these sorbents on native soil microbial communities remains unclear. Research has shown that the direct application of these materials (e.g. biochar, zeolite, red mud) may alter microbial community structure (Pietikäinen *et al.*, 2000; Garau *et al.*, 2007; Ahmad *et al.*, 2016), and thus, the use of soil amendments may impact microbial community function, biodegradation potential and ecosystem health.

Clay and modified clays are potential sorbents for remediating both metal and organic contaminants from soil and sediment (Chaerun Siti *et al.*, 2005; Yuan *et al.*, 2013). In particular, bentonite and its organically modified products (i.e. organoclays) are useful materials for environmental remediation (de Paiva *et al.*, 2008; He *et al.*, 2014). However, different cationic surfactants often used for preparing organoclays have the potential to exert toxicological effects on microorganisms (Ying, 2006). This could limit the application of these organoclays because microbial degradation is an environmentally friendly, efficient and cost‐effective strategy for remediating contaminants, especially PAHs (Xiong *et al.*, 2008; Megharaj and Naidu, 2017). However, toxicity may vary depending on the type of surfactant utilized. For example, Arquad^®^ 2HT‐75‐modified bentonite (AB) was less toxic to soil (micro)organisms than hexadecyltrimethylammonium and octadecyltrimethylammonium‐modified counterparts (Sarkar *et al.*, 2013), but more detrimental than its surface‐tailored (modified) organoclay (fatty acid‐grafted Arquad^®^‐modified bentonite) (ABP) (Biswas *et al.*, 2016). The modified organoclay showed selective binding for Cd in a phenanthrene–Cd‐spiked soil (Biswas *et al.*, 2016). This clay‐based product also showed high biocompatibility to bacteria in aqueous suspensions (Mandal *et al.*, 2016) and in a field‐contaminated soil (Biswas *et al.*, 2015). In the case of the field‐contaminated soil, sorbent‐mediated PAH biodegradation highlighted the ‘real‐world’ application of this modified organoclay as a potential in situ remediation product (Biswas *et al.*, 2018). However, Biswas *et al. *(2018) observed that although the modified organoclay increased bacterial growth (e.g. by cell colony count numbers on agar media) compared with its parent clay (i.e. bentonite), PAH biodegradation was not significantly different. These contrasting results raise an interesting question whether the addition of such modified organoclays could alter (i) the broader microbial (e.g. bacteria and archaea) community profile or (ii) the ratio of respiration‐active and inactive microbial groups, which resulted in the observed biodegradation outcomes. The effect of organoclays on soil microbial communities is also unclear with either stimulating or inhibitory effects depending on the type of organoclays and microorganisms assessed, for example that was reported by Abbate *et al. *(2009). The presence of organoclays may reduce the metabolic activity of particular microbial groups, whilst stimulating the growth of others that are more adaptable to material supplements (Abbate *et al.*, 2013). In the aforemention, microbial metabolic profiles in organoclay‐amended compost were investigated using single redox indicator, which did not provide a compressive assessment of microbial health and vitality (Abbate *et al.*, 2013).

To understand the fate and function of microbial communities associated with PAH biodegradation and Cd resistance, a comprehensive assessment of soil microbial DNA, diversity and active respiration profiling is necessary. Coupling 16S rRNA sequence analysis and redox‐based respiration (active *vs.* inactive cells) of microbial populations in clay‐amended field‐contaminated soil would provide an approach for studying the effect of (un)modified clays on PAH‐degrading microbial communities (Bowsher *et al.*, 2019). As a consequence, the aim of this study was to evaluate the impact of a surface‐tailored organoclay and its parent clays on native microbial activity in a long‐term PAH/Cd‐contaminated soil. This was achieved using soil microcosms incubated over a 70 days time course period and the assessment of (i) microbial relative abundance and diversity using 16S rRNA and (ii) active respiration i.e., metabolically active microbial cells using fluorescence technology.

## Results

### Microbial DNA mass in soil

**Figure 1:**
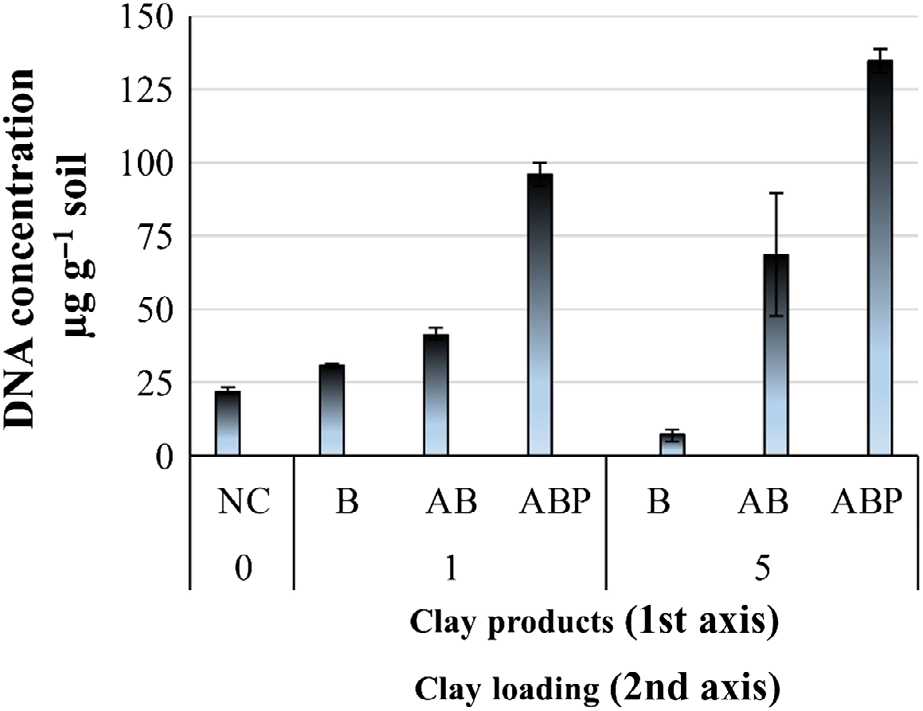
Soil DNA concentration following 70 days incubation of clay‐amended (B, bentonite; AB, Arquad^®^‐modified bentonite, ABP, palmitic acid‐modified AB) and unamended (NC) PAH/Cd co‐contaminated soil.

At the end of the 70 days incubation period, microbial DNA concentrations were significantly higher (*P* < 0.05) in soil supplemented with ABP compared with soil without clay amendments (NC hereafter), with unmodified clay (B) or organoclay (AB) (Fig. 1).

In the control (PAH/Cd‐contaminated soil without clay amendments), the concentration of soil DNA was 22.43 ± 1.04 µg g^−1^ soil. Similarly, the addition of B or AB to soil (clay loading 1%) resulted in significantly higher DNA concentrations compared with unamended soil (*P* < 0.05) (B = 30.82 ± 0.77 µg DNA g^−1^ soil; AB = 41.41 ± 2.01 µg DNA g^−1^ soil). However, in the presence of ABP, DNA of soil native microorganisms was three‐ to fourfold higher compared with the control and other clay‐treated soils (ABP = 95.73 ± 4.0 µg DNA g^−1^ soil) (*P* < 0.05). At a higher ABP loading (5%), DNA concentrations increased to 134.58 ± 3.90 µg DNA g^−1^ soil (*P* < 0.05), whereas when the parent product (AB, 5%) was assessed, there was no significant increase in DNA concentration (*P *> 0.05) (Fig. 1).

### Metabolically active microorganisms in clay‐amended soil

**Figure 2:**
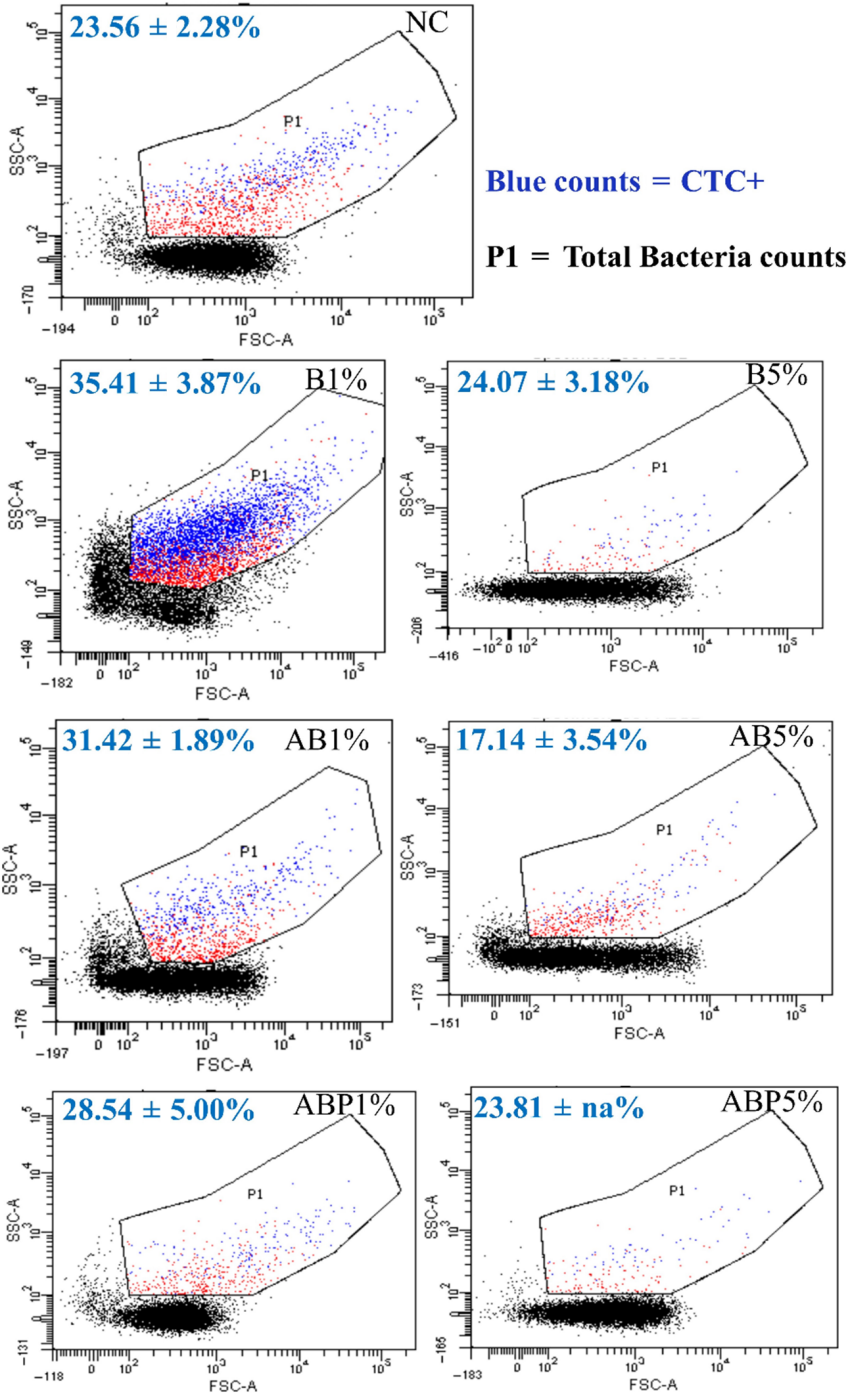
Total cell counts (P1 area) containing CTC‐positive (metabolically active) and inactive microbial cells in clay‐amended (1 and 5 % w/w of B, bentonite; AB, Arquad^®^‐modified bentonite, ABP, palmitic acid‐modified AB) and unamended (NC) PAH/Cd co‐contaminated soil. Blue counts (dots) represent CTC + cells, whilst red counts (dots) indicate inactive and/or dead cells. The per cent metabolically active cells of the total are expressed as Mean ± SD in each flow cytometry graph (*n* = 2, na = the duplicate replicate was destroyed during measurement). For the colour difference in the image, see the online version of this article.

Metabolic profiles indicate that the addition of clay and clay products at the lower loading (1%) increased metabolically active microbial cells (35.41 ± 3.87, 31.42 ± 1.89 and 28.54 ± 5.0% in B, AB and ABP‐amended soil, respectively, *vs.* 23.56 ± 2.28% in the control soil) (Fig. 2). However, when clay loading was increased to 5%, the percentage of active cells decreased irrespective of types of clay amendment (17.14–24.07% active cells). The addition of AB at 5% had the greatest impact on microbial metabolic activity (17.14 ± 3.54%), which was significantly lower than its counterparts (B and ABP) at the corresponding application rate (*P* < 0.05). In contrast, although lower, the metabolic impact of ABP at 5% was not significantly different (23.81% in ABP treatment) to the unamended control soil (Fig. 2).

### The relative abundance of soil microorganisms

**Figure 3:**
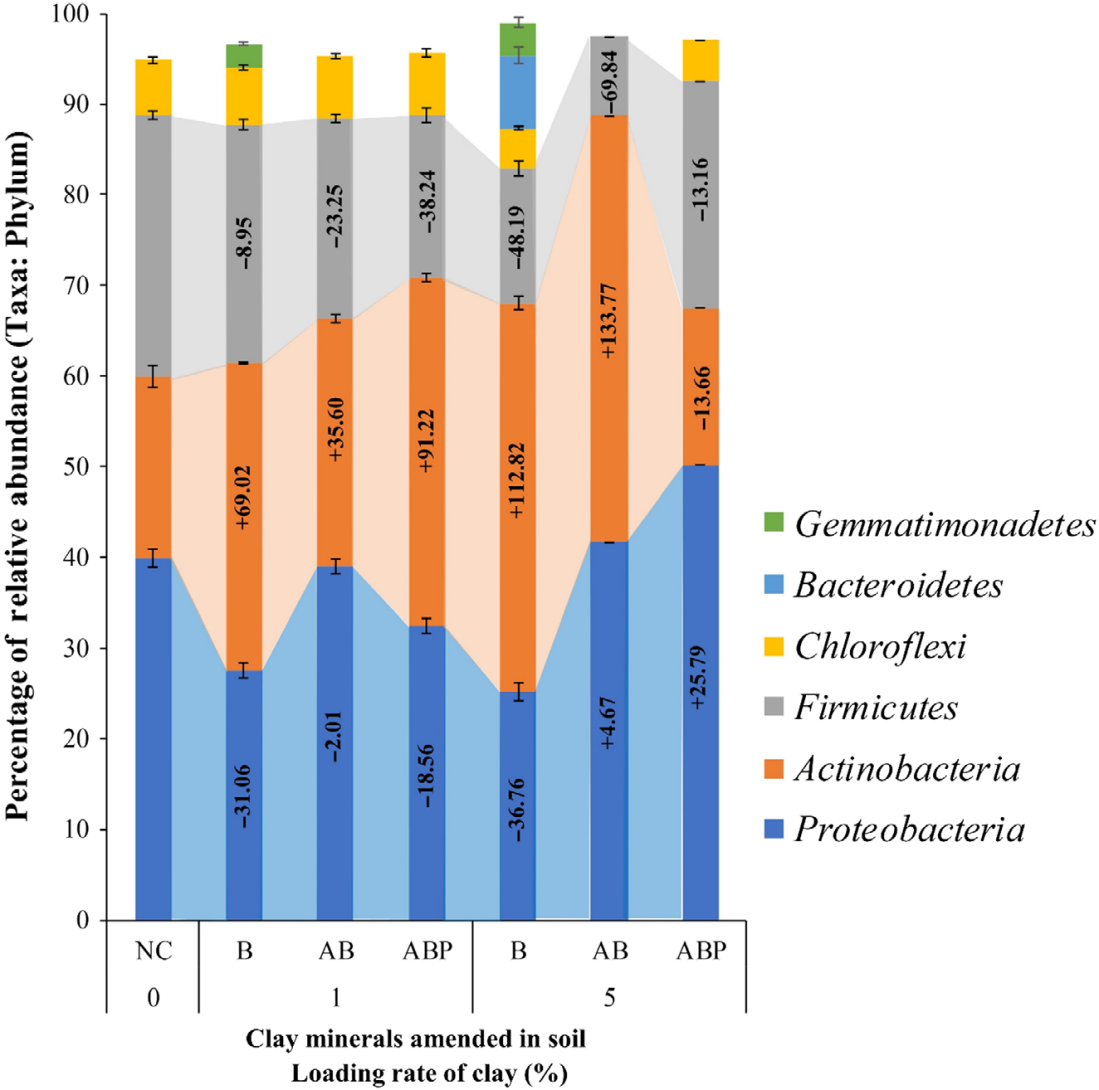
Relative abundance of microbial phyla in unamended (NC) and clay‐amended (B, bentonite; AB, Arquad^®^‐modified bentonite, ABP, palmitic acid‐modified AB) PAH/Cd co‐contaminated soil. The relative abundance of microorganism < 2% has been excluded from the graphical presentation. Each bar represents the Mean ± SD of triplicate analysis. The transparent area through the bars shows the trend of the three major taxa with the various clay products and their loading rates. Values on the bars of major phylum correspond to changes in relative abundance compared with the unamended control soil (NC).

Phylum with the relative abundance of > 2% will be reported in this section, whilst those less than that are listed in the Supporting Information (Fig. S1). The major bacterial taxa in the PAH/Cd‐contaminated field soil were *Actinobacteria*, *Proteobacteria*, *Firmicutes* and *Chloroflexi*, accounting for 94.84% of the total microbial population (archaea and unassigned bacteria accounted for 0.044%). Among these taxa, *Proteobacteria* was the dominant group (39.85 ± 0.10%) followed by *Firmicutes* (28.85 ± 0.43%), *Actinobacteria* (20.11 ± 1.24%) and *Chloroflexi* (6.04 ± 0.37). Whilst these groups contributed relative abundance of 94.84 ± 0.76%, the addition of B at the low dose (1%) increased the relative abundance to 96.60 ± 0.40% mainly by the addition of *Gemmatimonadetes*, which further increased (99.00 ± 0.70) at its higher loading (5%) by the contribution of *Bacteroidetes*. Although the application of AB and ABP did not increase the relative abundance of the total microbial population (i.e. values ranged between 95 and 97%), specific bacterial groups shifted significantly as a consequence of these soil amendments (*P* < 0.05). For example, *Actinobacteria* (33.99 ± 0.13%) was the dominant microbial group followed by *Proteobacteria* (27.47 ± 0.82%) and *Firmicutes* (26.27 ± 0.61%) when the raw clay (B, 1%) was used as the soil amendment (*P* < 0.05). The relative abundance of these groups changed (*Proteobacteria* (39.04 ± 0.81%) > *Actinobacteria* (27.27 ± 0.49%) > *Firmicutes* (22.14 ± 0.46%)) in the presence of AB1% (Fig. 3) (*P* < 0.05). In contrast, ABP induced a significant increase in *Actinobacteria* that shifted microbial abundance to *Actinobacteria* (38.45 ± 0.53%) > *Proteobacteria* (32.45 ± 0.76%) > *Firmicutes* (17.82 ± 0.85%) (*P* < 0.05).

When soil was amended with 5% clay products, the relative abundance of microbial groups was as follows (high to low): *Actinobacteria *> *Proteobacteria *> *Firmicutes* for B and AB whereas *Proteobacteria *> *Firmicutes *> *Actinobacteria* for ABP (*P* < 0.05). Among the other minor groups (Fig. 3), the presence of *Chloroflexi* was significantly impacted by the higher loading of AB (< 1.0% relative abundance in AB5% compared with 4.40–6.98% in the other treatments) (Fig. 3).

### Microbial OTU distribution, diversity and similarity in clay‐amended soils

**Figure 4:**
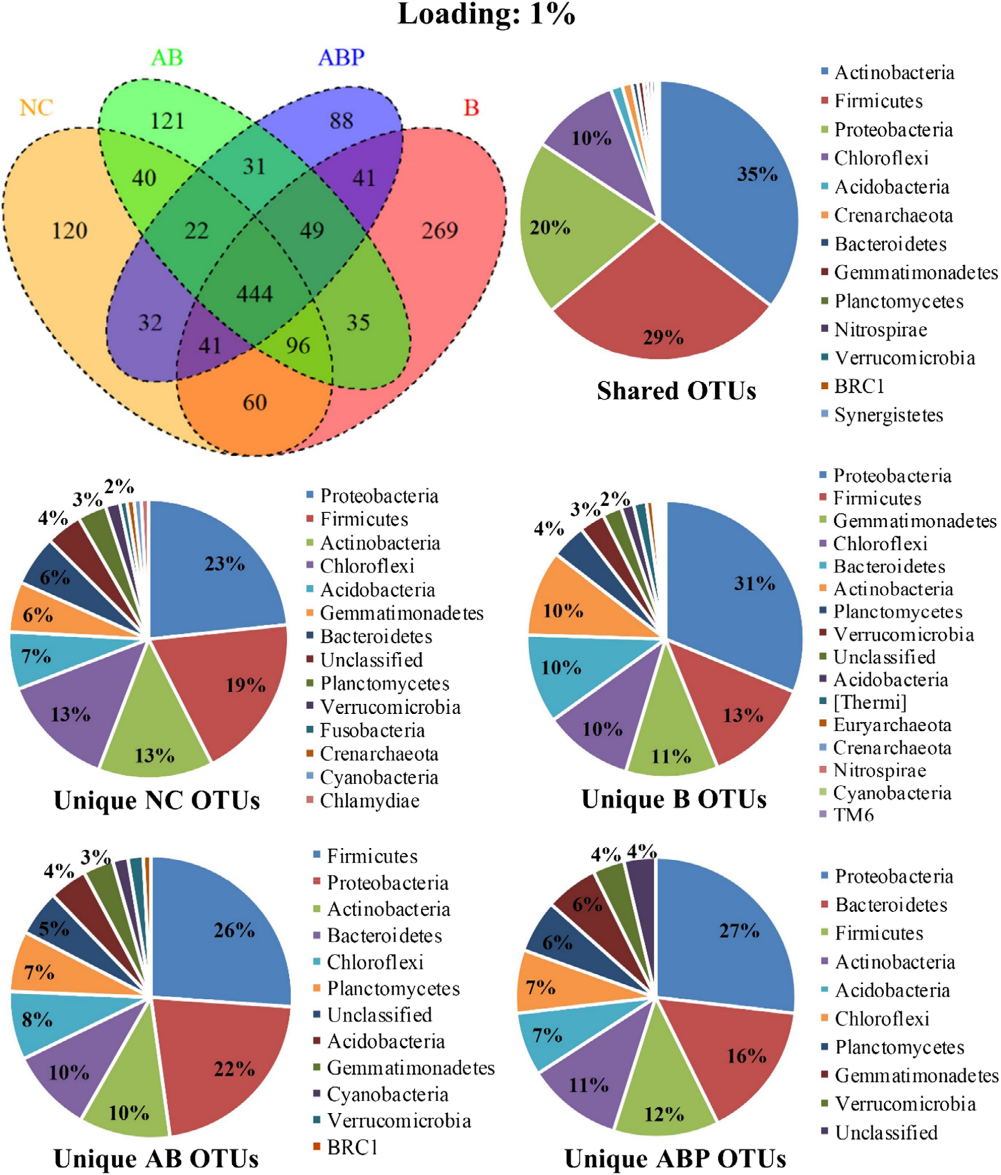
Venn diagram of OTUs (*n* = 3 replicates) in PAH/Cd co‐contaminated soil amended with 1% bentonite (B), Arquad^®^‐modified bentonite (AB), palmitic acid‐modified AB (ABP) or without clay amendments (NC). The percentage value was derived from the occurrence of OTUs corresponding to phylum within each pie chart.

**Figure 5:**
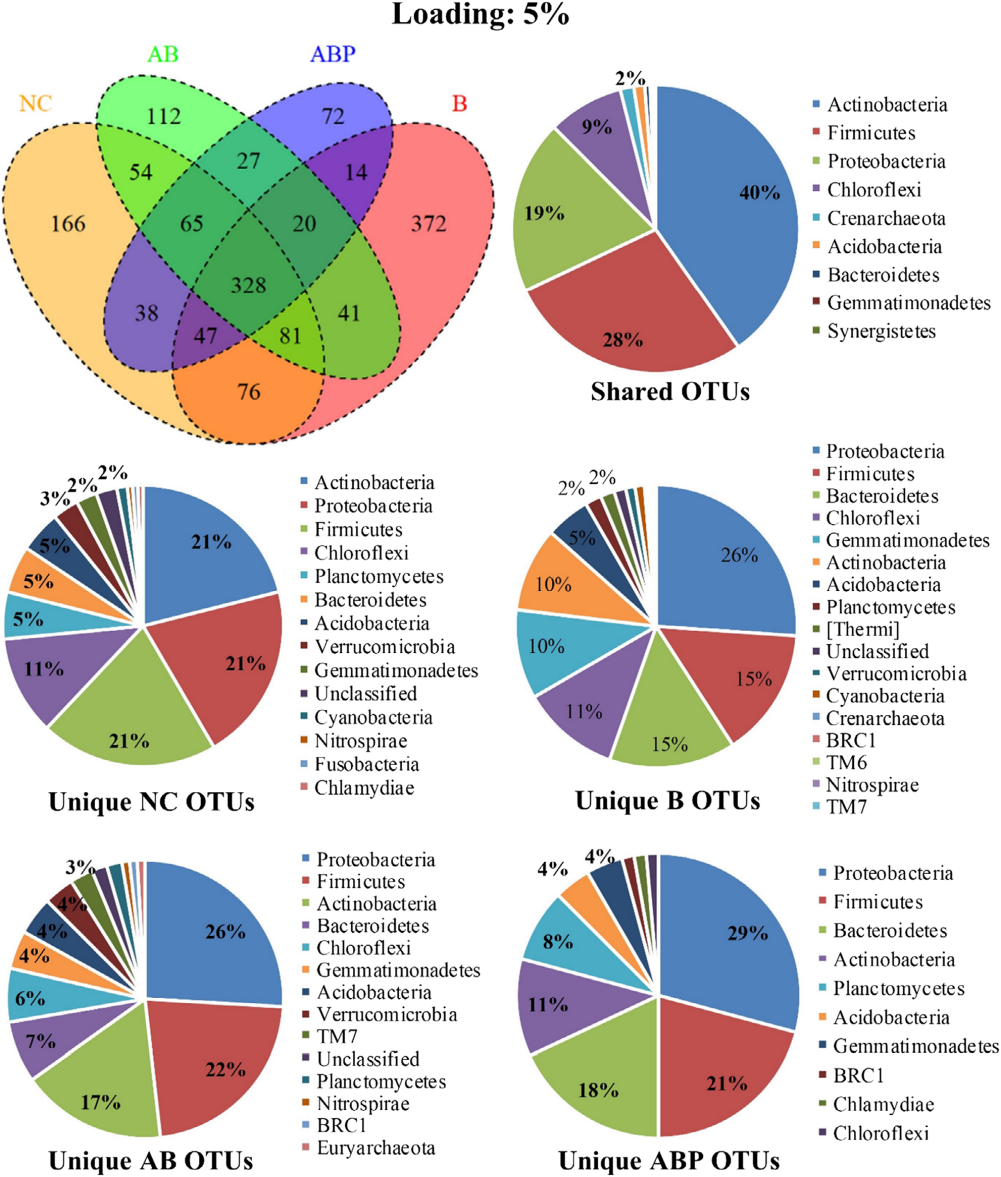
Venn diagram of OTUs (*n* = 3 replicates) in PAH/Cd co‐contaminated soil amended with 5% bentonite (B), Arquad^®^‐modified bentonite (AB), palmitic acid‐modified AB (ABP) or without clay amendments (NC). The percentage value was derived from the occurrence of OTUs corresponding to phylum within each pie chart.

A total of 24 phyla (22 bacteria and 2 archaea) were detected in the control and clay‐amended soils (total of seven samples with triplicates for each). Microbial phyla consisted of identified and unassigned 68 classes (~ 749 genera) with 1879 OTUs, which varied depending on the clay amendment applied into the soil (Figs 4 and 5). Unamended PAH/Cd‐contaminated soil hosted 855 OTUs, which increased to 1035 and 979 when B was applied (at 1% and 5% loading, respectively). However, the addition of AB and ABP at 1% decreased the number of OTUs to 838 and 748, respectively; an increased loading (5%) of these clay products further reduced OTUs to 728 and 611, respectively.

Of the 1879, 444 OTUs shared four treatments (unamended control, B, AB and ABP amendments at 1%), which was reduced to 328 when the material loading was increased to 5%. In all cases, *Actinobacteria, Firmicutes, Proteobacteria and Chloroflexi* were the major phyla available in all four soil treatments (Figs 4 and 5). The number of OTUs varied depending on the clay modification and loading. For example, at 1% loading, OTUs belonging to *Proteobacteria* were most frequent for all treatments except AB where *Firmicutes* were more prolific. Notably, another significant change in unique OTUs was those belonging to *Bacteroidetes* that appeared as the second‐ranked phylum after *Proteobacteria* in the ABP1%‐amended soil. When the ABP loading was increased to 5%, *Firmicutes* were more frequent compared with *Bacteroidetes,* whilst unclassified bacteria disappeared with the appearance of *Chloroflexi* and BRC.

**Figure 6:**
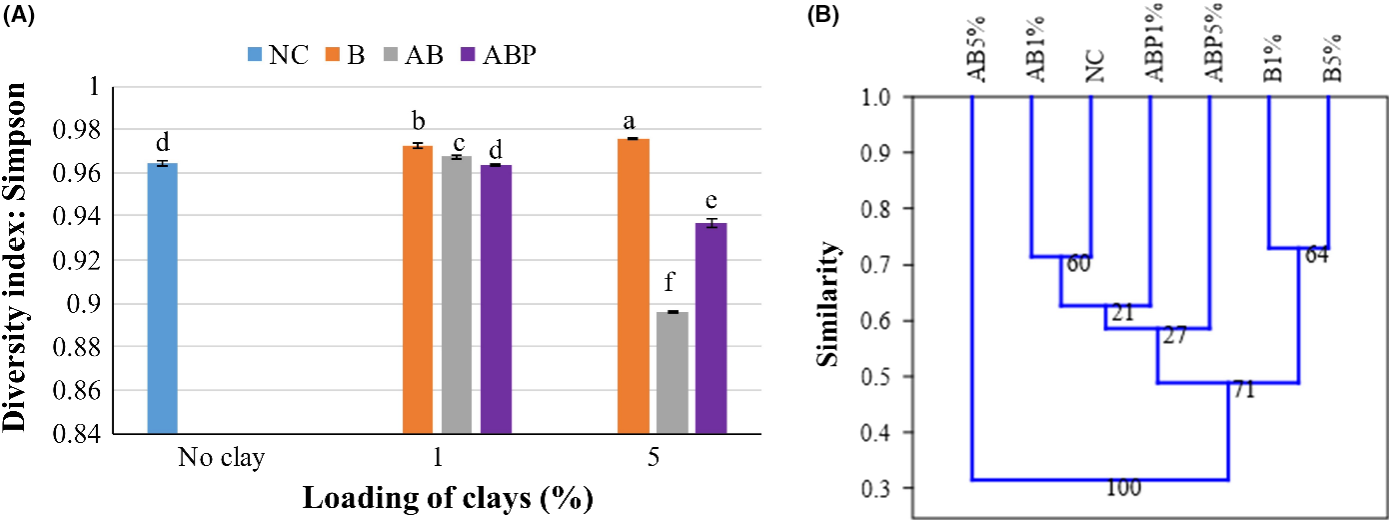
Diversity index (A) and UPGMA distant matrix (B) of unamended (control) and clay‐amended soils. NC = No clay (control), B = bentonite (raw clay), AB = Arquad‐bentonite (organoclay) and ABP = Arquad‐palmitic acid bentonite (modified organoclay). Loading rates (e.g. 1% or 5%) are expressed with the clay products, such as B1% is the expression of 1% of B loading. In the UPGMA, similarity matrix = Bray–Curtis, Bootstrapping Number = 1000. The number of OTUs was obtained as the mean of 3 replicates, and the bar on the ‘A’ graph shows SD of means. The different letters on each bar represent a significant difference among all treatments including loading rates at a 95% level of confidence using Duncan post hoc analysis.

Using OTUs, the similarity index following unweighted pair group method with arithmetic mean (UPGMA) indicated that the unamended control soil and the various clay‐amended soils appeared with distant microbial diversity profiles (Fig. 6). The addition of B1% increased bacterial OTU diversity (Simpson’s index, 0.973 ± 0.001) significantly higher than that in NC (0.964 ± 0.001) and to a greater extent when the loading was 5% (0.976 ± 0.0003) (*P* < 0.05). Although the addition of both organoclays (AB and ABP) reduced diversity, the impact of AB was higher with an increase in clay loading (AB5% = 0.896 ± 0.0003 *vs*. ABP5% = 0.937 ± 0.0002) (Fig. 6A). This diversity, relative abundance and habitat sharing of OTUs relate their distance in regard to treatments applied (Fig. 6B). For example, soil amended with AB5% was different compared with its application at 1%, whereas both ABP1% and ABP5% remained close to unamended (control) and the parent clay (B)‐amended soils (Fig. 6B).

## Discussion

### Effect of clay amendments on respiratory activity and microbial profile

During PAH biodegradation in soil, native microorganism profiles may undergo changes within the community structure as a consequence of the availability of carbon source over time, competition, etc. (Viñas *et al.*, 2005). However, changes may be further influenced as a result of changes in physicochemical properties arising from soil amendments applied to enhance bioremediation (Jung *et al.*, 2014; Al‐Kindi and Abed, 2016). The application of clay amendments to contaminated soil may alter microbial community structure (Cébron *et al.*, 2015), thereby influencing metabolic activity of soil microbial cells (as detailed in Fig. 2).

Whilst changes in microbial community profiles as a result of organoclay amendments has rarely been studied (Abbate *et al.*, 2009; Abbate *et al.*, 2013), there is a desire to study potential ecotoxicity caused by these materials due to their potential application for remediation purposes (Lazzara *et al.*, 2018). In our previous studies, the synthesized modified organoclay product (ABP) was identified as growth supportive for native heterotrophic bacteria in uncontaminated soil (Biswas *et al.*, 2015) and for *Mycobacterium gilvum* in an aqueous suspension containing phenanthrene (Mandal *et al.*, 2016). Those studies measured bacterial numbers and soil enzyme activity highlighting that microbial enzyme function might be impacted by conventional organoclay (e.g. AB) due to bacterial exposure to the cationic surfactant (e.g. Arquad^®^) from the clay surfaces and pores (Sarkar *et al.*, 2013). Arquad^®^ or related surfactants comprised quaternary ammonium compounds, containing nitrogen cations (N^+^) supported by four species of alkyl or aryl groups; they are the primary concern for toxicity to soil native microorganism (Li and Brownawell, 2010). Conceivably, these functional groups could exert toxicological impact on soil microorganisms (Reeve and Fallowfield, 2017) and inhibit biodegradation of hydrocarbon compounds (Ugochukwu *et al.*, 2014). However, the modified form of that organoclay (ABP) provided a congenial microhabitat for soil microorganism potentially through (i) anchoring of the exposed sites of the cationic surfactant through non‐toxic fatty acid molecules and (ii) creating a more hydrophobic clay mineral surface to facilitate solid surface‐supported microbial biofilms (Singh *et al.*, 2006; Leglize *et al.*, 2008). In the present study, we aimed to characterize microbial groups exposed to such organoclays and assess their health in clay‐amended soil. A significant finding of the study was that ABP amendment to PAH–Cd contaminated soil‐reduced microbial (e.g. bacteria and archaea) OTU numbers but not the major potential PAH‐degrading microbial groups (Figs 4 and 5). The comparative diversity index of microbial OTUs between amended soils further supports the biocompatibility of ABP over AB (Fig. 6). Extraction of gDNA at the end of the incubation period (day 70) from clay‐amended soil also indicated that ABP may support producing more DNA mass than raw bentonite. It is worth noting that the DNA extraction kit used in this study (see experimental section) extracts DNA from variety of organisms including bacteria, fungi and algae. Therefore, although it is partially relevance, our previous report on changes in bacterial colony forming unit (CFU) from the same soil and its amendments supports the present result (Biswas *et al.*, 2018) (Fig. S2).

### Implications for the remediation of PAH/Cd co‐contaminated soil

Material‐based amendments to suppress metal toxicity may be an important approach for the successful application of bioremediation in co‐contaminated soil. Kuppusamy *et al. *(2016) reported that metal‐tolerant and PAH‐degrading Gram‐negative bacteria such as those belonging to *Proteobacteria* were abundant in contaminated soil. In connection to the bioavailability of Cd reported in our previous study (Biswas *et al.*, 2018), we found that *Proteobacteria* were proportionally more abundant in the presence of Cd compared with the other two major groups, *Actinobacteria* and *Firmicutes* (Fig. 3 and Fig. S3). Indeed, Cd bioavailability in soil could be a significant determinant for the extent of metal toxicity on microorganisms (Thavamani *et al.*, 2015), and therefore, amendments that can bind Cd and other toxic metals may be useful to reduce microbial community impacts.

The soil used in this study contained a mixture of PAHs and Cd, where *Proteobacteria*, *Firmicutes, Actinobacteria* and *Chloroflexi* were the major bacteria (Fig. 3). Cadmium addition (and associated nitrate) to PAH‐contaminated soil may impact the microbial community structure (Lopez‐Fernandez *et al.*, 2018; Luo *et al.*, 2019), and however, these impacts are likely to be seen across all soil treatments as it was added shortly after the inclusion of clay materials. Using co‐contaminated soil (PAHs/metals such as Pb, Cr etc. in soil collected from coking plant wasteland, France; soil pH ~ 6.72–7.59, total organic carbon ~ 5.9%), Bourceret *et al. *(2016) reported that *Proteobacteria*, *Actinobacteria* and *Bacteroidetes* were dominant, whilst *Acidobacteria* was low in abundance. In this study, we also observed a relationship between *Proteobacteria* and *Actinobacteria,* although the abundance of each varied depending on the amendment applied. Reportedly, these bacterial groups are key degrader of PAHs in various types of oil‐contaminated soil and sludge (Bastiaens *et al.*, 2000; Viñas *et al.*, 2005; Isaac *et al.*, 2015).

### Effect of clay loading on microbial profiles and respiratory activity

Microbial relative abundance profiles changed by the addition of clay and modified clays to PAH/Cd‐contaminated soil but this depended on amendment loading. For example, at a low dose (1%), ABP‐amended soil had a high number of *Actinobacteria* and low count of *Proteobacteria*, which was opposite, whilst the dose was 5% of the same material (Fig. 3). The presence of a high abundance of PAH‐degrading *Proteobacteria* even at high applications (5%) of ABP may suggest that reportedly ‘toxic organoclays’ such as AB could be modified to reduce the toxicity of surfactant functional groups before application. Although surfactants are biodegradable in soil under aerobic conditions, cationic surfactants as quaternary ammonium compound (QAC) might be strong biocidal effect (Ying, 2006). Degradation or toxicity of such QAC depends on factors including alkyl chain length, position of quaternary ammonium in the carbon chain and loading concentration of surfactant per unit soil environment (Zhang *et al.*, 2015). Since AB was originally synthesized with a surfactant loading equivalent to 100% of bentonite cationic exchange capacity (Biswas *et al.*, 2016), it is expected that the surfactant was protected in the bentonite interlayer through cation exchange without significant leaching of surfactant molecules (Li *et al.*, 2003; Biswas *et al.*, 2019). The present study did not assess either the leaching potential of surfactants and palmitic acid molecules that were grafted into clays or biodegradation of them. This may warrant a further study. However, controlling similar conditions, the loading concentration of AB and ABP was comparable in both metabolic activity and microbial abundance where any potential exposure of surfactant was mitigated by palmitic acid in ABP. In this perspective, materials modified with surfactants or fatty acid might have the role player instead of unbound modifying compounds. For example, both ABP1%‐ and ABP5%‐amended soil provided habitats for microorganisms that were similar to control (NC) and raw clay‐mixed soil (see the distant matrix, Fig. 6B).

The redox‐based fluorescence stain marker, used to profile metabolic activities (active *vs.* inactive cells), revealed that with increasing modified clay load, CTC stain‐based metabolic rates were reduced, in particular for the cationic surfactant‐modified bentonite (AB) (Fig. 2). This is in contrast to the stimulatory effect on soil DNA concentration (Fig. 1), particularly for soil amended with the tailored organoclay (ABP). The effect was even more pronounced as clay amendment loading was increased (e.g. 5%). This contrasting phenomenon could be linked to the dormancy of soil native microorganism (Jones and Lennon, 2010) or carbon source‐specific microbial variability (Garland and Mills, 1991), which implies that (i) particular microbial groups utilized ABP‐amended soil to grow favourably but remained inactive specifically in the reduction‐based metabolism of CTC or similar compounds and/or (ii) the culturable microbial strains that grew well in the agar medium did not actively participate in the biodegradation of target compounds (Fig. S2).

## Conclusions and future implications

In a field soil contaminated with PAHs and Cd, clay and modified clay amendments altered 16S rRNA microbial profiles mainly among commonly reported PAH‐degraders (*Actinobacteria, Proteobacteria*, *Firmicutes* and *Chloroflexi*). Even at high dose (5%), the presence of *Proteobacteria* and *Actinobacteria* in soil amended with clay or modified clay may be advantageous for PAH biodegradation (Fuentes *et al.*, 2016). However, as revealed by redox‐fluorescence staining, metabolically active microbial cells were not as abundant in soil amended with modified organoclay compared with raw clay‐amended soil. Reducing the amendment load may improve microbial metabolic activity and minimize impacts such as reduced diversity. This is highly important in order to ensure the biocompatibility of modified clays for environmental remediation (Biswas *et al.*, 2019). A limitation of this study is that only 16S rRNA was used for the assessment of bacterial diversity, whilst the identification of functional genes responsible for PAH degradation or metal tolerance may provide greater insight in (organo)clay–microbial interactions and biodegradation impact (Liang *et al.*, 2019). Apart from bacteria and archaea, fungal community members are another significant group that may contribute to PAH degradation in co‐contaminated soil (Liu *et al.*, 2017). Further investigations considering these aspects and other environmental conditions, such as contrasting soil types and leaching of modifying agents from clays, would be beneficial to determine the impact of clay and modified clay addition of native soil microbial functionality.

## Experimental procedures

### Clay‐amended soil preparation and microcosm setup

**Figure 7:**
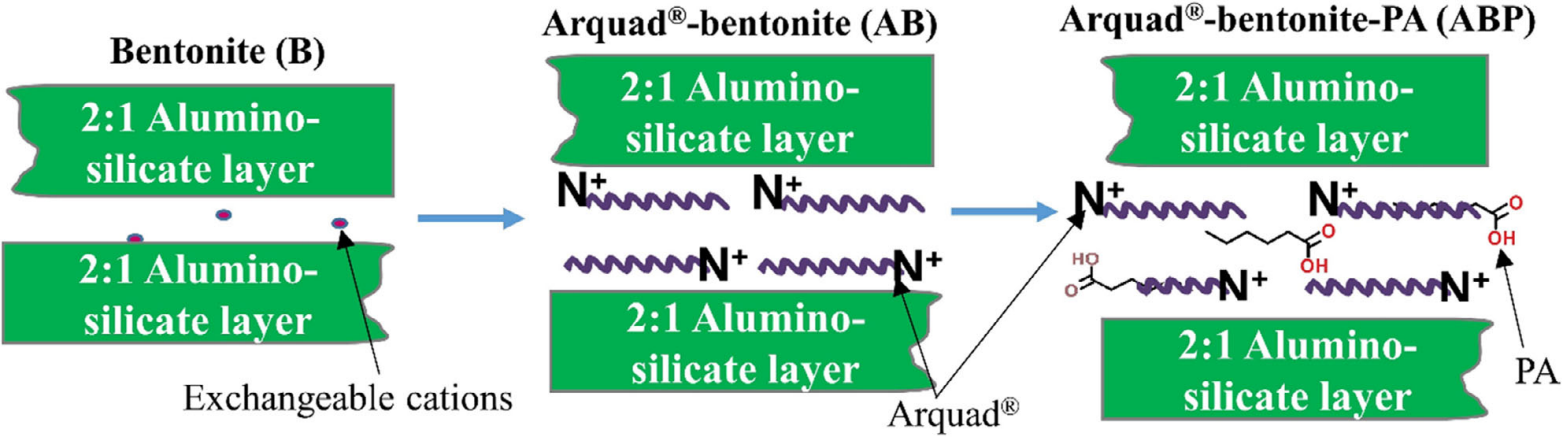
Schematic of the modified products (organoclay and surface‐tailored organoclay). The mineralogical and structural characteristics can be found elsewhere (Biswas et al., 2016; Mandal et al., 2016). PA, palmitic acid.

A loamy sand soil (pH = 6.4) with minimal clay content (4%) was received in mid‐2015 and stored at 4°C. Originally, it was collected from a mine site in South Australia (34.48 S, 138.37 E) (Juhasz *et al.*, 2014). The soil, containing a low fraction of clay (clay = 4%, silt = 8%, and sand = 88%, organic carbon = 2.1%), was chosen to minimize the effect of endemic clay content in order to assess the effect of clay addition into the soil. The soil was originally contaminated with PAH (∑PAHs = ~ 917 mg kg^−1^ soil). Briefly, soil (25 g) was mixed with clay products at (i) 1% and (ii) 5% w/w and agitated on an end‐over‐end shaker for 5 days. During this stage, soil was maintained at 30% water holding capacity. Three clay products were used as soil amendments; detailed characterization of bentonite (designated B) and its modified products, Arquad^®^‐bentonite (AB) and Arquad^®^‐palmitic acid‐treated bentonite (ABP) are described elsewhere (Biswas *et al.*, 2015, 2016; Mandal *et al.*, 2016) (Fig. 7).

Soil without clay addition served as the control treatment, named ‘NC’. Following clay addition, Cd(NO_3_)_2_ (150 mg Cd kg^−1^ soil) (> 99% purity, Chem‐supply, Australia) was added to the soil to effect a PAH/Cd co‐contaminated soil. Cadmium was spiked into amended soil and mixed for an additional 24 h after which the moisture content was increased and maintained at 60% WHC throughout the incubation period (70 days during the end of 2016). We utilized an incubation timeframe of 70 days based on the previous report where we stated that over this incubation period soil native bacteria has the potential to utilize available PAHs as a carbon and energy source fractions of PAHs variably depending on soil amendments; for example, ∑PAHs became ~ 402 mg kg^−1^ soil in the control soil (Biswas *et al.*, 2018). Soil sub‐samples were withdrawn from microcosms in triplicate in order to conduct various analyses as detailed in the following sections.

### Soil DNA concentration in clay‐amended soil

Following 70 days incubation, gDNA was extracted from soil samples (250 mg in triplicate) using the PowerLyzer^®^ PowerSoil^®^ DNA isolation kit (Mo Bio Laboratories, Inc., California, USA). Extracted DNA was quantified using fluorometer (Qubit^®^ 2.0, Invitrogen, Life technologies™, California, USA) and expressed as µg g^−1^ soil.

### Assessment of respiration‐active microbial cells using Flow Cytometry

#### Microbial cell isolation and CTC + staining

The metabolically functioning prokaryotic community was assessed using BacLight™ RedoxSensor™ Vitality kits (Molecular Probes™, Invitrogen, California, USA) following Whiteley *et al. *(2003). At the end of the incubation period (70 days), cells were isolated from soil (1 g) in duplicate. In brief, soil was added to sterile centrifuge tubes containing five sterile 5 mm‐sized glass beads and 4 ml phosphate buffer saline (PBS, pH = 7.4). Tubes were then shaken horizontally at 300 rpm to disperse cells from the soil matrix. After 1 h, samples were centrifuged (750 × g for 6 min at 4°C) to obtain microbial suspensions. Since fine soil particles and clay minerals are in the same size range of microbial cells, further separation with a gradient medium was necessary to obtain soil‐free cells (Amalfitano and Fazi, 2008). Supernatant (1.5 ml) were transferred onto 2 ml Eppendorf tubes containing non‐ionic density gradient medium Histodenz™ (400 µl of a 1.3 g ml^−1^) (Sánchez‐Andrea *et al.*, 2012). Tubes were centrifuged at 14 000 *g* for 30 min, and the Histodenz^TM^/PBS interface containing microbial cells (500 µl) was carefully withdrawn, transferred into another tube and diluted twofold with sterile 0.2 µm‐filtered PBS. Cell suspensions were stained with 5‐cyano‐2,3‐ditolyl tetrazolium chloride (CTC) at a final concentration of 5 mM. Briefly, a stock of CTC (25 mM) was prepared in 0.2 µm‐filtered water. Fresh cell suspensions (1000 µl) were mixed with 250 µl of the CTC stock to obtain a 5 mM CTC final concentration. Mixtures were then incubated in the dark for 2 h at room temperature (~ 23°C). To stop the reaction and to increase staining contrast, 1% paraformaldehyde (w/v) was added to suspensions. Controls were included to distinguish positive and null staining, including (i) sterile PBS with CTC stain and (ii) cell suspension without CTC addition.

#### Flow cytometry of CTC‐stained samples

Samples were assessed using a FACSCanto flow cytometer (BD Bioscience, California, USA). Microbial cells were gated on their light scatter properties, and CTC positivity was measured in the PerCp channel equipped with a 670 longpass filter at 488 nm.

### Soil microbial 16S rRNA diversity profiling

#### PCR and sequencing of amplicons

Following DNA extraction, PCR amplification and sequencing was performed by the Australian Genome Research Facility. 16S rRNA (V3–V4) amplicons were generated, and PCR was performed. The primers and PCR cycle conditions are presented in the SI 4. The resulting amplicons were sequenced on Illumina MiSeq (San Diego, CA, USA) with 2 × 300 base pairs paired‐end chemistry.

#### Sequencing assembling, diversity analysis and statistical interpretation

Paired‐end reads were assembled by aligning the forward and reverse reads using PEAR (v0.9.5) (Zhang *et al.*, 2014) followed by trimming of primers using seqtk (v1.0). Trimmed sequences were processed using Quantitative Insights into Microbial Ecology (QIIME 1.8) (Caporaso *et al.*, 2010), usearch (v7.1.1090) (Edgar, 2010; Edgar *et al.*, 2011) and UPARSE (Edgar, 2013) software. Throughout this process, quality filtering, removing full‐length duplicate sequences and sorting by abundance were performed. Singletons or unique reads in the data set were discarded. Sequences were clustered followed by chimera filtered using ‘rdp_gold’ database as the reference.

To obtain the number of reads in each OTU, reads were mapped back to OTUs with a minimum identity of 97%. Using QIIME, taxonomy was assigned using the silva r132 database (Quast *et al.*, 2012). These OTUs were sorted into available taxa, and the taxonomic level ‘phylum’ was used to produce relative abundance chart. The OTUs were taken to construct (i) distant matrix with unweighted pair group method with arithmetic mean (UPGMA) and similarity index ‘Bray–Curtis’ (Bootstrap N: 1000 using past3 software) and (ii) Venn diagram to identify the shared and distinct taxa and Simpson’s index for diversity analysis (using ‘VennDiagram’ and ‘Vegan’ package in R, respectively) (Chen, 2018; Oksanen *et al.*, 2019).

Analysis of variance (ANOVA) and post hoc analysis with Duncan’s multiple range test at 95% confidence level (*P* < 0.05) were applied to compare the mean of treatments by the use of imb spss Statistics 24 (New York, USA).

## Conflict of interest

None declared.

## Supporting information


**Fig. S1**. Relative abundance of microorganisms (Bacteria and Archea) which appeared to be less than 2% at phylum level after taxa annotation in SILVA database (r132). See experimental section in the main text for details.
**Fig. S2**. Bacterial growth (colony forming unit) (Left Y‐axis with Line) and microbial DNA mass (Right Y‐axis with Bar) in clay‐amended long‐term PAH/Cd‐contaminated soil. The CFU data have been replotted from our previous paper (Biswas et al., 2018) with the permission of Elsevier® 2018.
**Fig. S3**. The bioavailability of Cd in soil. The detail method of this experiment is found elsewhere (Biswas et al., 2018). The figure has been reused with the permission of Elsevier® 2018. The data presented in the inset table is the value for the day 70 only, extracted from the above figure. B, bentonite; AB, Arquad^®^‐modified bentonite, ABP, palmitic acid‐modified AB.
**Section SI4:** PCR and sequencing of amplicons.

## Data Availability

Quality‐filtered sequences have been submitted to NCBI archive and can be found under the Bioproject ID: PRJNA369284 and submission ID: SUB2358511.
