# Supplementary material for: Modified clays alter diversity and respiration profile of microorganisms in long‐term hydrocarbon and metal co‐contaminated soil
Source: Microb Biotechnol. 2019 Nov 11;13(2):522–34. doi: 10.1111/1751-7915.13510 (PMC7017831; doi:10.1111/1751-7915.13510)
Supplement: Supplementary file 1 — Fig. S1. Relative abundance of microorganisms (Bacteria and Archea) which appeared to be less than 2% at phylum level after taxa annotation in SILVA database (r132). See experimental section in the main text for details. Fig. S2. Bacterial growth (colony forming unit) (Left Y‐axis with Line) and microbial DNA mass (Right Y‐axis with Bar) in clay‐amended long‐term PAH/Cd‐contaminated soil. The CFU data have been replotted from our previous paper (Biswas et al., 2018) with the permission of Elsevier® 2018. Fig. S3. The bioavailability of Cd in soil. The detail method of this experiment is found elsewhere (Biswas et al., 2018). The figure has been reused with the permission of Elsevier® 2018. The data presented in the inset table is the value for the day 70 only, extracted from the above figure. B, bentonite; AB, Arquad®‐modified bentonite, ABP, palmitic acid‐modified AB. Section SI4: PCR and sequencing of amplicons. [file MBT2-13-522-s001.docx]

*Supplementary information* for-

**Modified clays alter diversity and respiration profile of microorganisms in long-term hydrocarbon and metal co-contaminated soil**

Bhabananda Biswas^a,b^^[[1]](#footnote-2)^, Albert L. Juhasz^a^, Mohammad Mahmudur Rahman^b,c^, Ravi Naidu^b,c^

^a^ Future Industries Institute, University of South Australia, Mawson Lakes, SA 5085, Australia

^b^ Cooperative Research Centre for Contamination Assessment and Remediation of the Environment (CRC CARE), ATC Building, The University of Newcastle, Callaghan, NSW 2308, Australia

^c^ Global Centre for Environmental Remediation (GCER), The University of Newcastle, Callaghan, NSW 2308, Australia

**
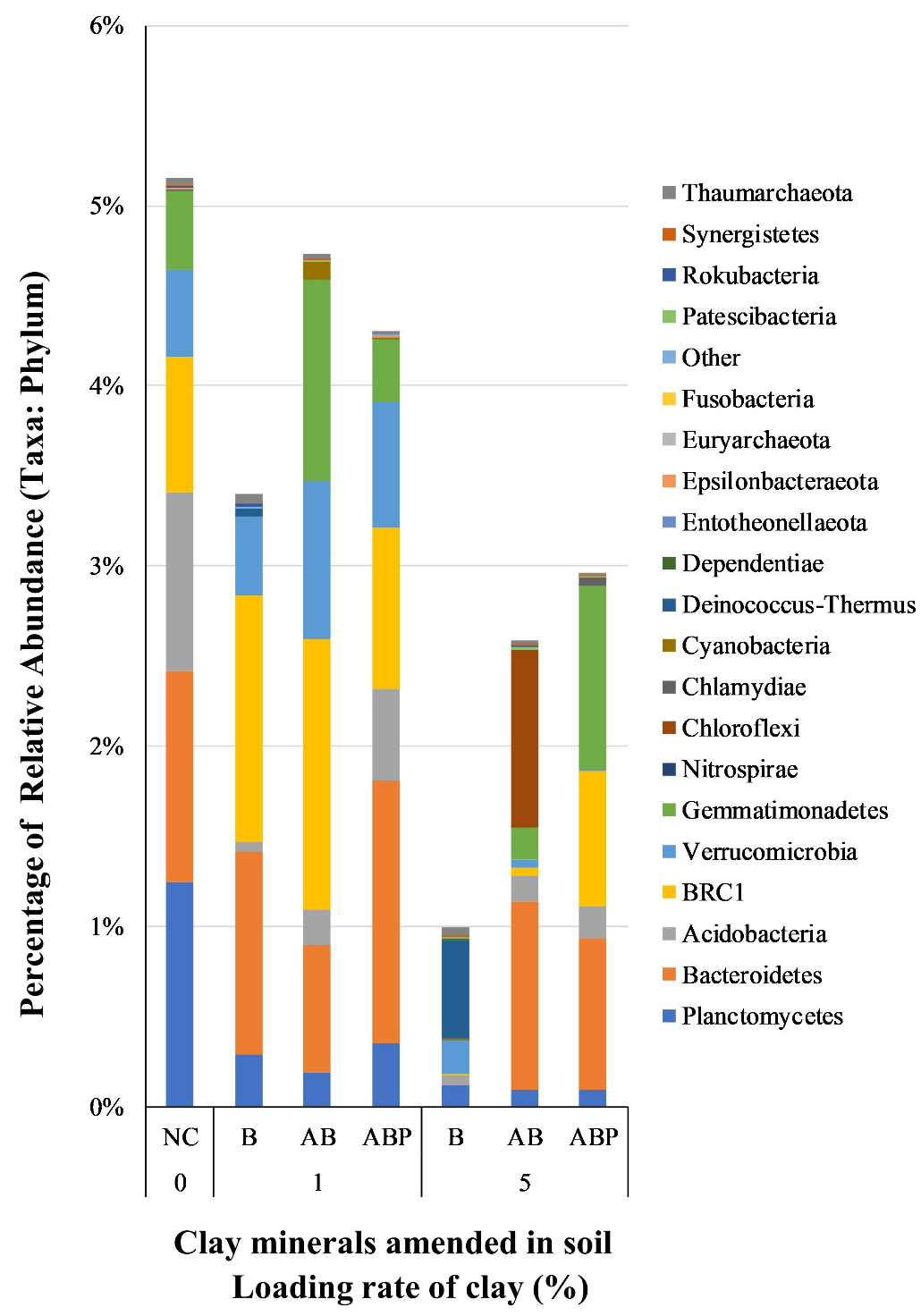
SI 1. Relative abundance (<2%) of microorganisms**

**Fig. S1** Relative abundance of microorganisms (Bacteria and Archea) which appeared to be less than 2% at phylum level after taxa annotation in SILVA database (r132). See experimental section in the main text for details.

**SI 2. Bacterial growth and soil microbial DNA concentration in soil**

(See main text for the description of soil incubation, genomic DNA extraction from soil). For bacterial colony-forming unit (CFU) study, a solution (10 mL) of sodium hexametaphosphate (35 g L^−1^) and sodium carbonate (7 g L^−1^) was used as the dispersing medium of the incubated soil (1 g) (Pascaud et al., 2012). This solid-liquid mixture was kept at vigorous shaking on an orbital shaker at 300 rpm overnight, which allowed the bacteria to be dispersed and homogeneously distributed in the mixture. The bacterial growth was measured by counting CFU on plates containing nutrient agar media after 3–5 days of incubation at 25 °C.


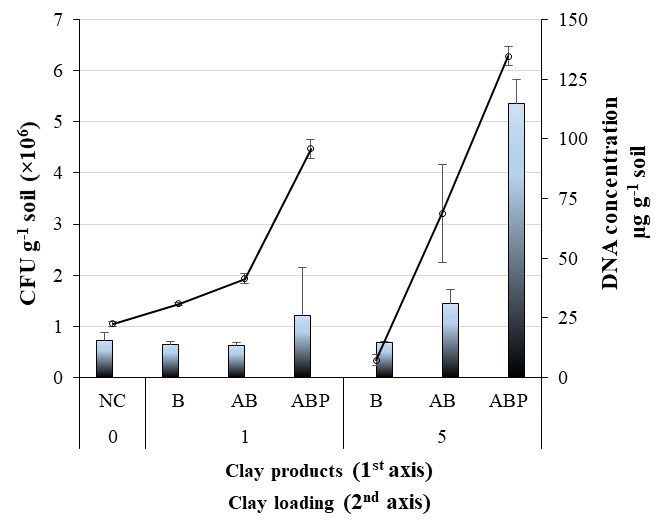


Fig. S2 Bacterial growth (colony forming unit) (Left Y-axis with Line) and microbial DNA mass (Right Y-axis with Bar) in clay-amended long-term PAH/Cd-contaminated soil. The CFU data have been replotted from our previous paper (Biswas et al., 2018) with the permission of Elsevier® 2018.

In the control soil (PAH/Cd-contaminated but without clay amended), the growth of bacteria was found 7.3 × 10^5^ ± 1.47 × 10^5^ CFU g^-1^ (22.43 ± 1.04 µg DNA g^-1^ soil). At this period, either raw bentonite or the organobentonite (AB) supplemented soil (clay loading 1%) changed the bacterial count and soil DNA concentration (B = 6.62 × 10^5^ ± 0.53 × 10^5^ CFU g^-1^ and 30.82 ± 0.77 µg DNA g^-1^ soil; AB = 6.16×10^5^ ± 0.61×10^5^ CFU g^-1^ and 41.41 ± 2.01 µg DNA g^-1^ soil. In the presence of the modified organoclay (ABP), the growth and loading of the native bacteria appeared two- to fourfold higher of the control and other clay-treated soils (ABP = 12×10^5^ ± 9.5×10^5^ CFU g^-1^ and 95.73 ± 4.0 µg DNA g^-1^ soil). At a higher loading rate (5%) of ABP, this growth even increased up to 53.7×10^5^ ± 4.7×10^5^ CFU g^-1^ and 134.58 ± 3.90 µg DNA g^-1^ soil whereas its parent product (AB) rather remained stable in the growth of soil bacteria (Fig. S2).

**SI 3 Cadmium (Cd) availability in soil**

Fig. S3 The bioavailability of Cd in soil. The detail method of this experiment is found elsewhere (Biswas et al., 2018). The figure has been reused with the permission of Elsevier® 2018. The data presented in the inset table is the value for the day 70 only, extracted from the above figure. B, bentonite; AB, Arquad^®^-modified bentonite, ABP, palmitic acid-modified AB.

**SI 4 PCR and sequencing of amplicons**

16S-rRNA (V3–V4) amplicons were generated using the primers 341F (5´–CCTAYGGGRBGCASCAG–3´) and 806R (5´–GGACTACNNGGGTATCTAAT–3´) using AmpliTaq Gold 360 mastermix (Life Technologies™, Australia) for the primary PCR. The PCR cycle conditions were: 7 min at 95 °C, followed by 29 cycles of 30 s at 94 °C, 1 min at 50 °C (ramp 3 °C/s), 1 min at 72 °C and final elongation time 7 min at 72 °C. A secondary PCR to index the amplicons was performed with TaKaRa Taq DNA Polymerase (Takara Bio USA, Inc.). The resulting amplicons were measured by fluorometry (Quant-iT™ PicoGreen^®^ dsDNA assay kit, Invitrogen) and normalized. The equimolar pool was then measured by qPCR (KAPA Biosystems, USA) followed by sequencing on Illumina MiSeq (San Diego, CA, USA) with 2 × 300 base pairs paired-end chemistry.

**References**

Biswas, B., Sarkar, B., Faustorilla, M.V., and Naidu, R. (2018) Effect of surface-tailored biocompatible organoclay on the bioavailability and mineralization of polycyclic aromatic hydrocarbons in long-term contaminated soil. *Environ Technol Innov* **10**: 152-161.

Pascaud, A., Soulas, M.-L., Amellal, S., and Soulas, G. (2012) An integrated analytical approach for assessing the biological status of the soil microbial community. *Eur J Soil Biol* **49**: 98-106.

1. Corresponding author: email: [Bhaba.Biswas@unisa.edu.au](mailto:Bhaba.Biswas@unisa.edu.au); [B.Nanda.Biswas@gmail.com](mailto:B.Nanda.Biswas@gmail.com); p: +61 08 83025181 [↑](#footnote-ref-2)
